# Design, Synthesis, and Evaluation of Novel Phenolic Acid/Dipeptide/Borneol Hybrids as Potent Angiotensin Converting Enzyme (ACE) Inhibitors with Anti-hypertension Activity

**DOI:** 10.3390/molecules22111739

**Published:** 2017-11-03

**Authors:** Ying Sun, Yujun Bai, Xirui He, Yajun Bai, Pei Liu, Zefeng Zhao, Xufei Chen, Xiaohui Zheng

**Affiliations:** 1 Key Laboratory of Resource Biology and Biotechnology in Western China, Ministry of Education, Northwest University, Xi’an 710069, China; syqsn1108@163.com (Y.S.); tianleyu@163.com (Y.B.); 6105194@163.com (X.H.); baiyj@nwu.edu.cn (Y.B.); lp183929@163.com (P.L.); zzf598155752@sina.com (Z.Z.); m13085333798@163.com (X.C.); 2 Key Laboratory of Synthetic and Natural Functional Molecule Chemistry of the Ministry of Education, College of Chemistry & Materials Science, Northwest University, Xi’an 710127, China

**Keywords:** phenolic acid/dipeptide/borneol hybrid, ACE inhibitor, hypertension, combination of traditional Chinese medicine chemistry

## Abstract

Under the guidance of combination of traditional Chinese medicine chemistry (CTCMC), this study describes the preparation of a phenolic acid/dipeptide/borneol hybrid consisting of phenolic acid and a bornyl moiety connected to the dipeptide *N*-terminal and *C*-terminal respectively. It also evaluates their angiotensin converting enzyme (ACE) inhibitory and synergistic antihypertensive activities. Briefly, a series of novel phenolic acid/dipeptide/borneol hybrids were prepared and investigated for their ability to inhibit ACE. The influence of the phenolic acid and bornyl moiety on subsite selectivity is also demonstrated. Among all the new compounds, two compounds—**7a** and **7g**—reveal good inhibition potency in in vitro ACE-inhibitory tests. Interestingly, favorable binding results in molecular docking studies also supported the in vitro results. Additionally, the bioassay showed that oral administration of the two compounds displayed high and long-lasting antihypertensive activity both in acute antihypertensive tests and in therapeutic antihypertensive tests by non-invasive blood pressure measurements in spontaneously hypertensive rats.

## 1. Introduction

Hypertension is a metabolic cardiovascular syndrome that affects nearly one billion people around the world [1,2]. Clinically, it is the major risk factor for various heart diseases, such as arteriosclerosis, coronary heart disease, stroke, peripheral arterial disease, and heart failure [3,4]. Nowadays, the angiotensin converting enzyme (ACE) inhibitors (e.g., Capoten (captopril), Vasotec (enalapril), Prinivil, or Zestril (lisinopril), etc.) have been considered as first-line drugs for the treatment of hypertension and myocardial infarction [5,6]. However, it was found that any single currently used ACE inhibitor cannot completely prevent and cure hypertension. Clinically, they are usually used in conjunction with one or two other therapeutic agents including beta blockers, long-acting nitrate, and calcium channel blockers for a better therapeutic effect (e.g., Enalapril/hydrochlorothiazide, Enalapril/Valsartan, and Enalapril/nifedipine, etc.). Moreover, ACE inhibitors are also found to have some side effects, such as cough, elevated blood potassium levels, low blood pressure, and kidney failure [7,8]. Therefore, the trend has been toward developing safer and multi-functional ACE inhibitors.

**Figure 1:**
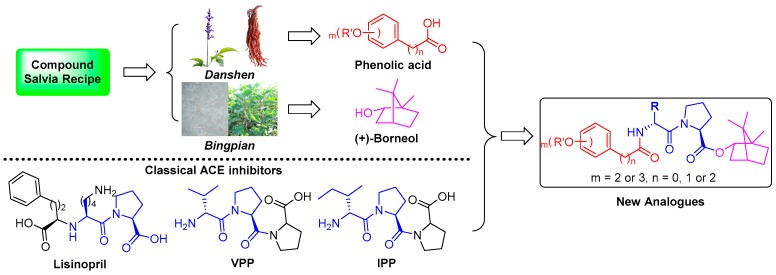
Structure design inspired by the formula of compound *Salvia* recipe and classical angiotensin converting enzyme (ACE) inhibitors. VPP: valine-proline-proline; IPP: isoleucine-proline-proline.

Many pioneering work in the mid-1970s to mid-1990s revealed that the *N*-substituted derivative of proline is an extremely potent ACE inhibitor [9,10,11,12]. Recently, during our study on compound *Salvia* recipe (including three Chinese herbal medicines: *Salvia miltiorrhiza* Bge. (Danshen), *Panax pseudoginseng* Wall. (Sanqi), and Borneol (Bingpian, from *Cinnamomum camphora* (L.) Presl)) for searching new antihypertensive drugs, we noticed that the phenolic acids extract from Danshen plays a pivotal role in regulating blood pressure, protecting against oxidative damage diseases, slightly slowing the heart rate, and relaxing and widening blood vessels [13], while borneol may help other medicinal molecules to penetrate cell membrane easily [14,15]. In fact, the molecular dimension of some phenolic acids, especial those structures varying from C_6_–C_1_ to C_6_–C_3_, are more suitable for ACE S_1_ subsite [16] and the hydroxyls on these phenolic acids may serve as good hydrogen donors to chelate with Zn^2+^ in the active site of ACE [17]. More recently, some phenolic acids, such as ferulic acid, gallic acid, chlorogenic acid, and caffeic acid, have been demonstrated to be a new class of selective inhibitors of ACE [18,19]. So, in this study, based on the effectiveness of compound *Salvia* recipe in the prevention and treatment of angiocardiopathy and the ideas of CTCMC, we tried to add these effective phenolic acid and bornyl moieties simultaneously to the *N*-terminal and *C*-terminal of dipeptides (Val or Leu-Pro, the core structure of some lead ACE inhibitors [20,21,22]) (see Figure 1) by simply using amide and ester bonds, respectively. We envision that this molecular construction strategy may result in producing more novel lead compounds with multi-functions, not only targeting ACE, but also other targets relating to synergistic hypotensive effects, which can overcome some side effects of current ACE inhibitors.

## 2. Materials and Methods

### 2.1. Synthesis and Preparation of ***7a**–**7h***

**Scheme 1:**
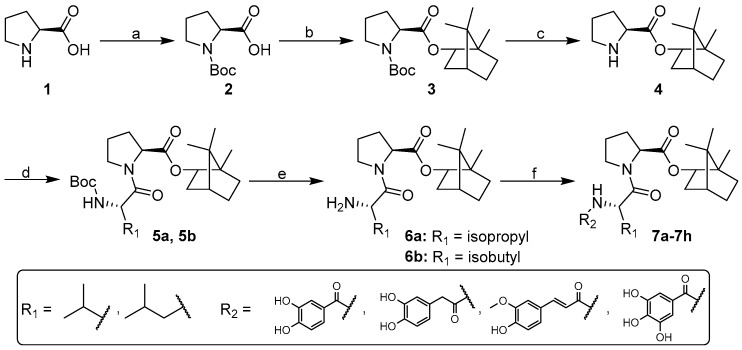
Synthesis of new ACE Inhibitors **7a**–**7h**. Reagents, conditions, and yields: (**a**) (Boc)O_2_, dioxane/H_2_O, NaOH, 0 °C–25 °C, 12 h, 86% isolated yield; (**b**) EDCI, DMAP, (+)-borneol, THF, room temperature, 18 h, 75% isolated yield; (**c**) TFA:DCM = 1:5, room temperature, 6 h, 91% isolated yield; (**d**) EDCI, HOBT, corresponding Boc-protective amino acid, DMF, room temperature, 10–20 h; (**e**) TFA:DCM = 1:4, room temperature, 4–8 h; (**f**) corresponding phenolic acids, Pybop, DIPEA, THF, DMF or DMSO, room temperature, under nitrogen atmosphere, 12–30 h.

Commercially available l-proline **1** was initially converted to *N*-Boc-Proline **2** in the presence of di-*tert*-butyl dicarbonate in good yield and then followed by bornyl ester formation under Steglich esterification conditions to afford *N*-Boc-Pro-bornyl ester **3**. This was subjected to trifluoracetic acid(TFA)/dichloromethane(DCM) conditions to afford compound **4**, which was coupled with *N*-Boc-Val and *N*-Boc-Leu respectively under the 1-ethyl-3-(3-dimethyllaminopropyl) carbodiimide hydrochloride(EDCI)/1-hydroxybenzotriazole (HOBT) system to furnish the corresponding *N*-Boc-dipeptide ester **5a** and **5b**. Removal of the Boc group of **5a** and **5b** was accomplished with the above mentioned TFA/DCM conditions to give **6a** and **6b** in near-quantitative yields. Finally, the target compounds **7a**–**7h** were achieved by general coupling reaction of **5a** and **5b** with corresponding phenolic acids under the benzotriazole-1-yl-oxytripyrrolidinophosphonium hexafluorophosphate(Pybop)/*N*,*N*-diisopropyl-ethylamine (DIPEA) system. The synthesis of the new ACE inhibitors **7a**–**7h** is shown in Scheme 1.

### 2.2. Biological Investigations

#### 2.2.1. In Vitro ACE-Inhibitory Activity

The in vitro ACE-inhibitory activity of the newly synthesized compounds was evaluated using ACE inhibition assay (Sigma-aldrich, St. Louis, MO, USA, No. A-6778). The experiments were carried out for all the designed compounds (**7a**–**7h**), for ACE-inhibitory activity at five different concentrations (0.01–10 µM). Lisinopril, a well-known ACE inhibitor drug, was used as standard for comparison with the inhibitory activity of synthesized compounds. The ACE-inhibitory activity can be calculated by the following equation: ACE-inhibitory activity (inhibition rate %) = [(A_blank1_ − A_sample_)/(A_blank1_ − A_blank2_)] × 100, where blank 1: Positive control (without ACE inhibition) and blank 2: Reagent blank. Then we measured and calculated the IC_50_ values of the designed compounds (**7a**–**7h**). 

#### 2.2.2. Molecular Docking Study 

To gain insight into the interaction of ACE with the most potent compound, it was docked on to tACE (PDB code: 1O86) using AutoDock Vina software (v4.2) (The Scripps Research Institute, San Diego, CA, USA). Structures of the ligand were generated using ChemBioDraw Ultra (v13.0) (Cambridgesoft, Billerica, MA, USA).

#### 2.2.3. Antihypertensive Tests In Vivo

All the experiments were approved by the Institutional Animal Ethics Committee of Northwest University. Experiments were carried out on spontaneously hypertension rats (SHRs) that were 9–10 weeks old and weighed 200–220 g. SHRs treated with normal saline were used as controls. Lisinopril was used as a standard drug. Blood pressure (BP) was recorded automatically every day by the indirect tail-cuff method using an electrosphygmomanometer (BP-2010A Storage Pressure Meter). In acute antihypertensive tests in vivo, the most potent and active compounds obtained in in vitro tests were measured using spontaneously hypertensive rats. The BP were measured before and 0.5, 1, 2, 3, 4, 5 and 6 h after a single dose of intragastrical administration of active compounds. In therapeutic antihypertensive tests, the more potent and active compound and lisinopril were treated orally for 7 consecutive days, and the BP was measured every day between drug delivery day and 14 days after the drug treatment using an intelligent non-invasive blood pressure remote monitoring instrument.

## 3. Results and Discussion

### 3.1. Chemistry

**Scheme 2:**
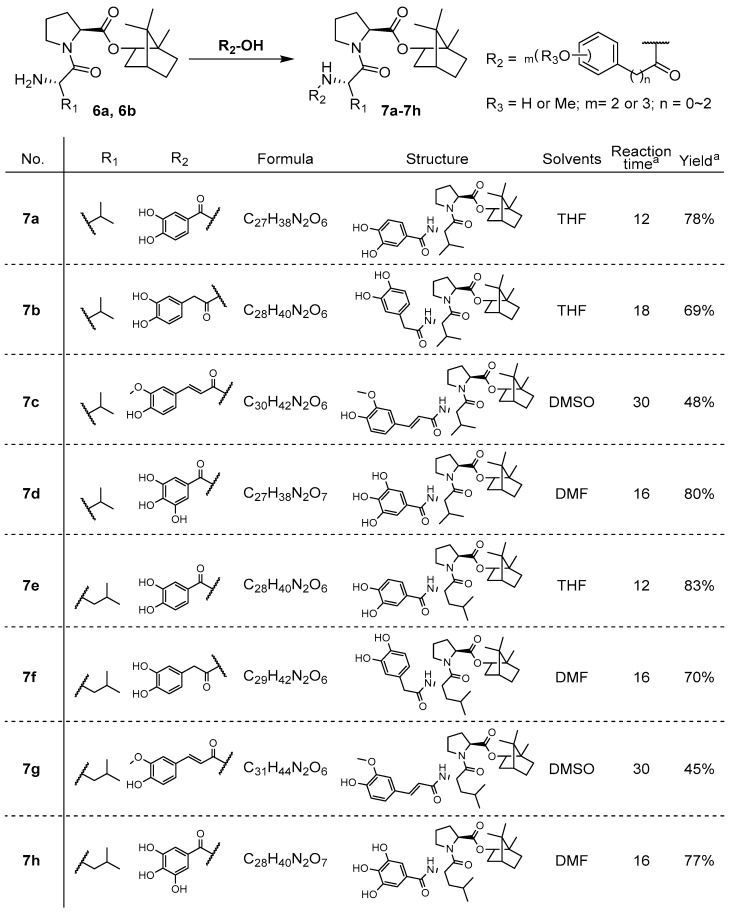
Chemical structure, synthetic conditions and yield of **7a**–7**h**. ^a^ For the last step.

The synthesis of target compounds **7a**–**7h** was achieved following the procedures outlined in Scheme 1. The chemical structure, synthetic conditions and yield of **7a**–**7h** are shown in Scheme 2. 

#### Compound Identification (**7a**–**7h**)

(**7a**). White powder. *m*/*z* = [M + 1] 487.2804, calculated value: 486.2729. ^1^H-NMR (600 MHz, CDCl_3_) δ 8.34 (s, 1H), 7.40 (dd, *J* = 30.8, 9.5 Hz, 1H), 7.31 (s, 1H), 7.18 (d, *J* = 8.4 Hz, 1H), 6.80 (d, *J* = 8.3 Hz, 1H), 4.98 (d, *J* = 9.9 Hz, 1H), 4.72 (t, *J* = 8.1 Hz, 1H), 4.46 (dd, *J* = 8.4, 4.6 Hz, 1H), 4.02–3.94 (m, 1H), 3.78–3.70 (m, 1H), 2.34–2.30 (m, 1H), 2.25–2.17 (m, 3H), 2.08–2.01 (m, 2H), 2.00–1.93 (m, 2H), 1.92–1.85 (m, 1H), 1.77–1.70 (m, 1H), 1.31–1.22 (m, 3H), 1.12 (d, *J* = 6.7 Hz, 3H), 1.04 (d, *J* = 6.7 Hz, 3H), 0.99 (d, *J* = 6.7 Hz, 1H), 0.97–0.95 (m, 1H), 0.89 (s, 3H), 0.86 (s, 3H), 0.81 (s, 3H). ^13^C NMR (151 MHz, CDCl_3_) δ 172.22 (s), 171.83 (s), 168.25 (s), 148.42 (s), 144.02 (s), 125.46 (s), 120.53 (s), 115.10 (s), 114.29 (s), 81.03 (s), 59.57 (s), 56.76 (s), 49.09 (s), 48.13 (s), 44.91 (s), 36.46 (s), 31.47 (s), 29.83 (s), 29.48 (s), 28.04 (s), 27.21 (s), 24.94 (s), 19.81 (s), 19.51 (s), 18.93 (s), 18.63 (s), 13.74 (s).

(**7b**). White powder. *m*/*z* = [M + 1] 501.2970, calculated value: 500.2886. ^1^H-NMR (600 MHz, CDCl_3_) δ 7.41 (s, 1H), 6.99 (s, 1H), 6.68 (d, *J* = 12.4 Hz, 1H), 6.62–6.51 (m, 2H), 4.94 (d, *J* = 9.8 Hz, 1H), 4.55 (t, *J* = 8.1 Hz, 1H), 4.52–4.43 (m, 1H), 3.99–3.93 (m, 1H), 3.70–3.57 (m, 1H), 3.49–3.44 (m, 1H), 3.37 (d, *J* = 15.5 Hz, 1H), 2.33–2.26 (m, 1H), 2.24–2.19 (m, 1H), 2.09–1.94 (m, 5H), 1.89–1.84 (m, 1H), 1.79–1.67 (m, 2H), 1.35–1.15 (m, 3H), 1.03 (d, *J* = 6.6 Hz, 3H), 0.91 (d, *J* = 7.3 Hz, 3H), 0.87 (s, 3H), 0.85 (s, 3H), 0.79 (s, 3H). ^13^C-NMR (151 MHz, CDCl_3_) δ 173.05 (s), 172.12 (s), 171.41 (s), 125.94 (s), 121.52 (s), 116.33 (s), 115.93 (s), 115.49 (s), 81.01 (s), 59.58 (s), 56.40 (s), 49.03 (s), 48.09 (s), 47.75 (s), 44.87 (s), 42.75 (s), 36.46 (s), 31.29 (s), 29.50 (s), 28.04 (s), 27.20 (s), 24.99 (s), 19.80 (s), 19.41 (s), 18.91 (s), 18.37 (s), 18.02 (s), 13.71 (s).

(**7c**). White powder. *m*/*z* = [M + 1] 527.3128, calculated value: 526.3042. ^1^H-NMR (600 MHz, CDCl_3_) δ 8.22 (s, 1H), 7.51 (d, *J* = 15.5 Hz, 1H), 7.01 (d, *J* = 8.2 Hz, 1H), 6.98 (s, 1H), 6.89 (d, *J* = 8.1 Hz, 1H), 6.53 (d, *J* = 8.4 Hz, 1H), 6.29 (d, *J* = 15.5 Hz, 1H), 5.03–4.94 (m, 1H), 4.89–4.80 (m, 1H), 4.80–4.70 (m, 1H), 4.59–4.51 (m, 1H), 3.90 (dd, *J* = 16.2, 3.9 Hz, 3H), 3.77–3.69 (m, 1H), 3.67–3.55 (m, 1H), 2.36–2.30 (m, 1H), 2.26–2.21 (m, 1H), 2.18–2.11 (m, 1H), 2.10–2.01 (m, 3H), 1.94–1.88 (m, 1H), 1.78–1.72 (m, 1H), 1.71–1.66 (m, 1H), 1.29–1.24 (m, 2H), 1.10 (d, *J* = 6.6 Hz, 3H), 0.96 (d, *J* = 6.7 Hz, 3H), 0.89 (s, 3H), 0.87 (s, 3H), 0.81 (s, 3H). ^13^C-NMR (151 MHz, CDCl_3_) δ 172.31 (s), 171.14 (s), 166.24 (s), 147.61 (s), 146.91 (s), 141.44 (s), 127.39 (s), 122.63 (s), 118.13 (s), 114.85 (s), 109.48 (s), 80.73 (s), 59.29 (s), 56.02 (s), 49.08 (s), 48.10 (s), 47.49 (s), 44.92 (s), 36.49 (s), 31.73 (s), 29.50 (s), 28.07 (s), 27.22 (s), 25.03 (s), 19.82 (s), 18.93 (s), 18.10 (s), 13.74 (s).

(**7d**). White powder. *m*/*z* = [M + 1] 503.2755, calculated value: 502.2679.^1^H-NMR (600 MHz, CDCl_3_) δ 8.34 (s, 1H), 7.46 (s, 1H), 7.48 (s, 1H), 7.33 (s, 2H), 5.00 (d, *J* = 9.8 Hz, 1H), 4.73 (t, *J* = 8.1 Hz, 1H), 4.48 (dd, *J* = 8.4, 4.6 Hz, 1H), 4.00–3.93 (m, 1H), 3.76–3.69 (m, 1H), 2.36–2.31 (m, 1H), 2.25–2.19 (m, 3H), 2.08–2.00 (m, 2H), 1.95–1.90 (m, 2H), 1.90–1.83 (m, 1H), 1.79–1.73 (m, 1H), 1.29–1.21 (m, 3H), 1.16 (d, *J* = 6.8 Hz, 3H), 1.08 (d, *J* = 6.8 Hz, 3H), 0.99 (d, *J* = 6.8 Hz, 1H), 0.98–0.95 (m, 1H), 0.91 (s, 3H), 0.87 (s, 3H), 0.80 (s, 3H). ^13^C-NMR (151 MHz, CDCl_3_) δ 172.21 (s), 171.84 (s), 168.27 (s), 148.45 (s), 148.47 (s), 144.00 (s),120.58 (s), 114.31 (s), 81.02 (s), 59.55 (s), 56.78 (s), 49.06 (s), 48.17 (s), 44.90 (s), 36.44 (s), 31.43 (s), 29.85 (s), 29.44 (s), 28.02 (s), 27.25 (s), 24.97 (s), 19.85 (s), 19.52 (s), 18.94 (s), 18.65 (s), 13.75 (s).

(**7e**). White solid. *m*/*z* = [M + 1] 501.2970, calculated value: 500.2886. ^1^H-NMR (600 MHz, CDCl_3_) δ 8.41 (s, 1H), 7.45 (s, 1H), 7.31 (s, 1H), 7.18 (d, *J* = 8.3 Hz, 1H), 6.81 (d, *J* = 8.3 Hz, 1H), 5.03–4.98 (m, 1H), 4.55 (dd, *J* = 8.6, 4.3 Hz, 1H), 3.72–3.62 (m, 1H), 3.53–3.38 (m, 1H), 3.33–3.21 (m, 1H), 2.41–2.34 (m, 1H), 2.24–2.19 (m, 1H), 2.00–1.92 (m, 2H), 1.91–1.84 (m, 1H), 1.82–1.70 (m, 2H), 1.68–1.50 (m, 3H), 1.30–1.18 (m, 3H), 1.00 (d, *J* = 6.5 Hz, 3H), 0.93 (d, *J* = 6.5 Hz, 3H), 0.88 (s, 3H), 0.87 (s, 3H), 0.80 (s, 3H). ^13^C-NMR (151 MHz, CDCl_3_) δ 174.01 (s), 172.09 (s), 171.88 (s), 148.43 (s), 144.00 (s), 125.43 (s), 120.50 (s), 115.13 (s), 114.27 (s), 81.19 (s), 59.37 (s), 49.04 (s), 48.09 (s), 47.24 (s), 44.99 (s), 41.12 (s), 39.95 (s), 36.70 (s), 29.30 (s), 28.17 (s), 27.33 (s), 24.85 (s), 24.71 (s), 23.51 (s), 21.48 (s), 19.83 (s), 18.93 (s), 13.74 (s).

(**7f**). White solid. *m*/*z* = [M + 1] 515.3125, calculated value: 514.3042. ^1^H-NMR (600 MHz, CDCl_3_) δ 7.40 (s, 1H), 6.98 (s, 1H), 6.64 (d, *J* = 12.2 Hz, 1H), 6.60–6.50 (m, 2H), 5.01–4.98 (m, 1H), 4.55 (dd, *J* = 8.4, 4.1 Hz, 1H), 3.71–3.65 (m, 1H), 3.53–3.37 (m, 1H), 3.35–3.22 (m, 1H), 2.36–2.29 (m, 1H), 2.27–2.17 (m, 1H), 2.06–1.97 (m, 2H), 1.91–1.83 (m, 1H), 1.81–1.70 (m, 2H), 1.69–1.53 (m, 3H), 1.33–1.21 (m, 3H), 1.02 (d, *J* = 6.4 Hz, 3H), 0.96 (d, *J* = 6.7 Hz, 3H), 0.88 (s, 3H), 0.86 (s, 3H), 0.80 (s, 3H). ^13^C-NMR (151 MHz, CDCl_3_) δ 173.04 (s), 172.15 (s), 171.42 (s), 125.95 (s), 121.50 (s), 116.31 (s), 115.92 (s), 115.47 (s), 80.84 (s), 59.46 (s), 56.42 (s), 49.02 (s), 48.09 (s), 47.63 (s), 44.83 (s), 42.51 (s), 36.52 (s), 29.93 (s), 29.12 (s), 27.26 (s), 24.97 (s), 24.78 (s), 22.60 (s), 21.87 (s), 19.20 (s), 18.98 (s), 13.72 (s).

(**7g**). Light yellow solid. *m*/*z* = [M + 1] 541.3273, calculated value: 540.3199. ^1^H-NMR (600 MHz, CDCl_3_) δ 8.18 (s, 1H), 7.47 (d, *J* = 15.6 Hz, 1H), 6.98 (d, *J* = 8.2 Hz, 1H), 6.94 (s, 1H), 6.87 (d, *J* = 8.2 Hz, 1H), 6.27 (d, *J* = 15.6 Hz, 1H), 5.00–4.95 (m, 1H), 4.56 (dd, *J* = 8.5, 4.2 Hz, 1H), 3.89 (s, 3H), 3.70–3.64 (m, 1H), 3.54–3.39 (m, 1H), 3.36–3.20 (m, 1H), 2.38–2.29 (m, 1H), 2.26–2.19 (m, 1H), 2.07–1.99 (m, 2H), 1.93–1.84 (m, 1H), 1.83–1.71 (m, 2H), 1.71–1.54 (m, 3H), 1.34–1.20 (m, 3H), 1.03 (d, *J* = 6.5 Hz, 3H), 0.95 (d, *J* = 6.7 Hz, 3H), 0.89 (s, 3H), 0.87 (s, 3H), 0.81 (s, 3H). ^13^C-NMR (151 MHz, CDCl_3_) δ 172.22 (s), 171.80 (s), 166.28 (s), 147.61 (s), 146.92 (s), 141.35 (s), 127.44 (s), 122.58 (s), 118.12 (s), 114.88 (s), 109.59 (s), 80.74 (s), 59.26 (s), 56.02 (s), 49.05 (s), 48.09 (s), 47.03 (s), 44.93 (s), 42.21 (s), 36.62 (s), 29.43 (s), 28.12 (s), 27.29 (s), 24.98 (s), 24.88 (s), 23.60 (s), 21.87 (s), 19.80 (s), 18.91 (s), 13.72 (s).

(**7h**). White solid. *m*/*z* = [M + 1] 517.2924, calculated value: 516.2835. ^1^H-NMR (600 MHz, CDCl_3_) δ 8.35 (s, 1H), 7.48 (s, 1H), 7.47 (s, 1H), 7.32 (s, 2H), 5.00–4.96 (m, 1H), 4.55 (dd, *J* = 8.3, 4.1 Hz, 1H), 3.71–3.66 (m, 1H), 3.55–3.41 (m, 1H), 3.36–3.27 (m, 1H), 2.35–2.25 (m, 1H), 2.23–2.18 (m, 1H), 2.05–1.98 (m, 2H), 1.97–1.86 (m, 1H), 1.84–1.75 (m, 2H), 1.72–1.53 (m, 3H), 1.35–1.22 (m, 3H), 1.02 (d, *J* = 6.6 Hz, 3H), 0.97 (d, *J* = 6.6 Hz, 3H), 0.91 (s, 3H), 0.87 (s, 3H), 0.81 (s, 3H).^13^C-NMR (151 MHz, CDCl_3_) δ 174.21 (s), 171.81 (s), 166.27 (s), 148.45 (s), 148.47 (s), 144.00 (s), 121.60 (s), 120.58 (s), 114.31 (s), 80.68 (s), 59.25 (s), 56.12 (s), 49.02 (s), 48.05 (s), 47.00 (s), 44.91 (s), 42.31 (s), 36.66 (s), 29.42 (s), 28.11 (s), 27.30 (s), 24.98 (s), 24.89 (s), 23.60 (s), 21.85 (s), 19.82 (s), 18.89 (s), 13.71 (s).

### 3.2. ACE-Inhibitory Activity In Vitro

**Figure 2:**
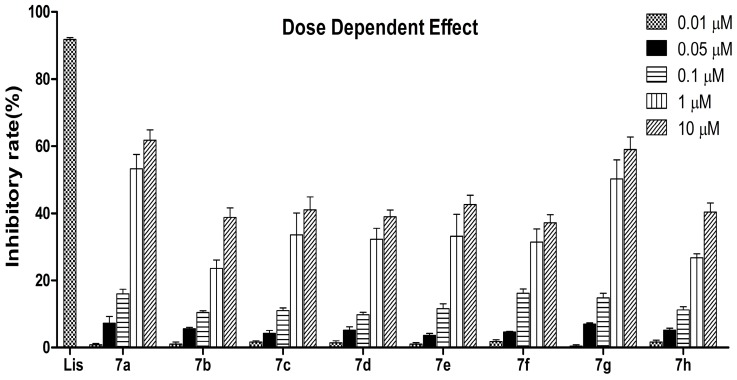
Dose-dependent angiotensin converting enzyme (ACE) inhibition activity of test compounds and lisinopril (Lis). The results are presented as means ± SEM (*n* = 8).

The preliminary biological investigations were conducted in vitro using the ACE-inhibition assay. The experiments carried out for all the designed compounds (**7a**–**7h**), for ACE-inhibitory activity at five different concentrations (0.01–10 µM), revealed that these newly synthesized compounds inhibited ACE in a dose-dependent manner (Figure 2). The evaluation results of all eight new compounds for their ACE-inhibitory activity are presented in Table 1. Unfortunately, all of these new compounds showed a comparatively lower ACE-inhibitory activity. Relatively, evaluation of IC_50_ data against ACE for these compounds revealed that **7a** and **7g** are the best candidates and more encouraging compounds with the lowest IC_50_ values of 6.76 and 6.88 μM, respectively. In contrast, the size of the amino acid side chain had a great influence on the activity of these compounds. That is, the activity of these compounds gradually declined when the size of the amino acid side chain increased. Besides, the influence of phenolic acids on ACE-inhibitory activity was more obvious following the order: protocatechuic acid > ferulic acid > gallic acid > 3,4-dihydroxybenzene acid. That is, substituents in positions 3 and 4 in the benzene ring are beneficial to the increase of ACE-inhibitory activity. At the same time, the shorter the amino side chain in phenolic acids, the better the ACE-inhibitory activity.

### 3.3. ACE Molecular Docking

**Figure 3:**
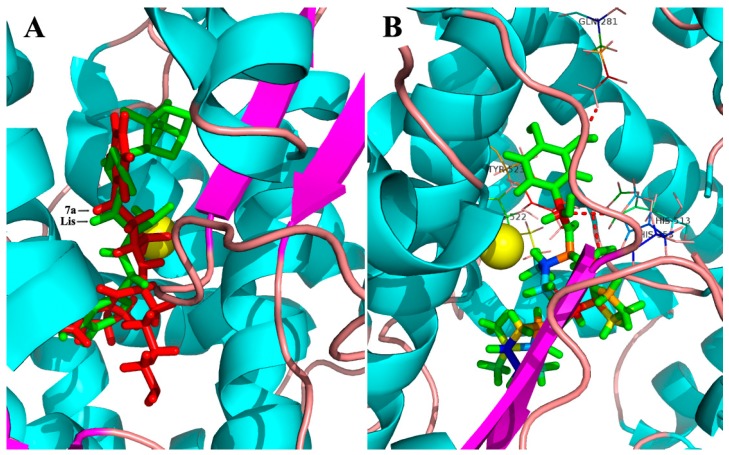
(**A**) Docking confirmation of compound **7a** (green) and ligand compound lisinopril (red) with tACE; 1, α-helix; 2, random coil; 3,β-sheet; (**B**) Best expected binding mode of compound **7a** (green) at the tACE binding site.

Based on the inhibition results, we selected the most potent compound **7a**, our best ACE inhibitor in this study, as a ligand example. To gain insight into the interaction of ACE with compound **7a**, it was docked on to tACE (PDB code: 1O86) using AutoDock Vina software [23]. As shown in Figure 3A, **7a** and ligand compound (Lisinopril) of tACE can nearly overlap in the binding model. That is, they have the same binding sites and have a similar combination model. The intermolecular interactions between tACE binding site residues and predicted poses for compound **7a** are presented in Figure 3B. Results showed that the docking position of compound **7a** showed a similar binding mode to that of lisinopril. The intermolecular hydrogen-bonding interactions of 7a were mainly seen with the Gln281, His 353, His 513, Tyr 522 and Tyr 523, which are partly similar to lisinopril. 

### 3.4. Antihypertensive Tests In Vivo

**Figure 4:**
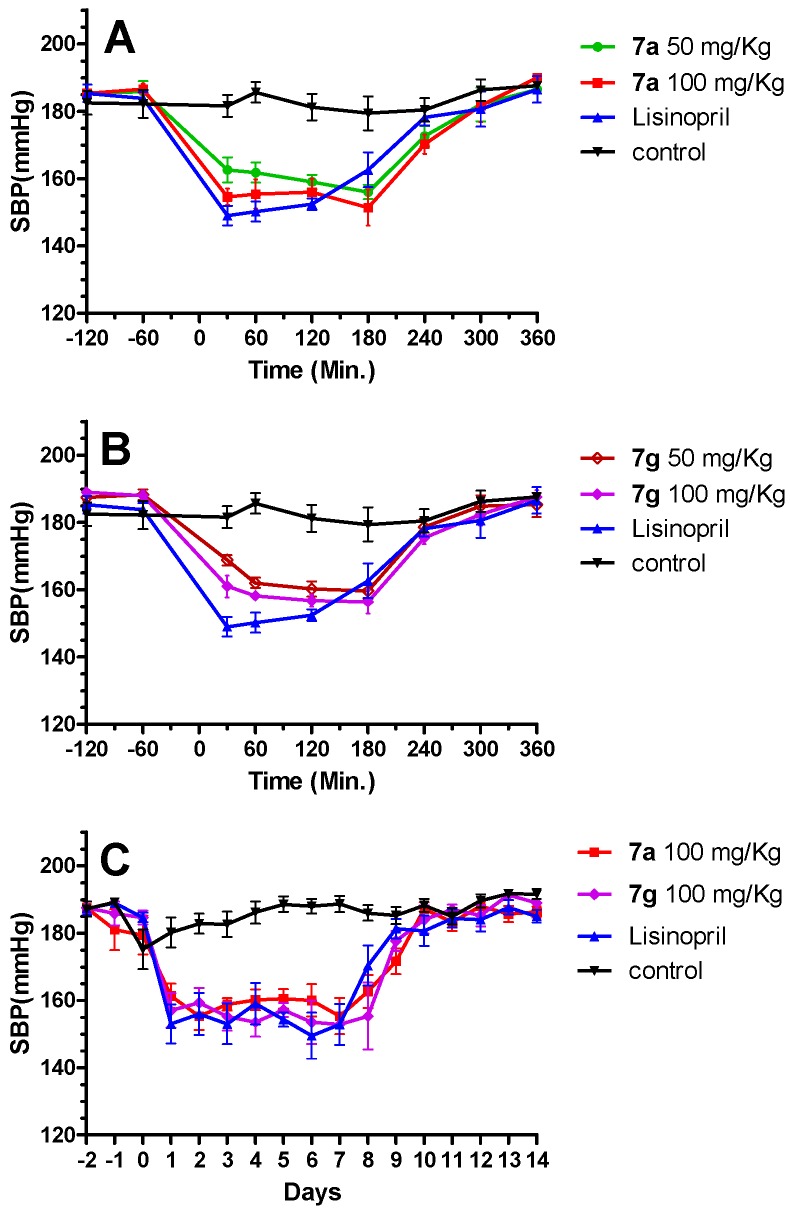
(**A**,**B**), the acute antihypertensive activity of compounds **7a** and **7g** in spontaneously hypertension rats (SHRs) for single dose administration (dosing time: 0 min); (**C**) the therapeutic antihypertensive activity of compounds **7a** and **7g** in SHRs for 7 days treatment (dosing time: 0 day; blood pressure (BP) measured time: 9:30–11:30 am). Data are presented as mean ± SEM, *n* = 6. The BP was determined with a BP-2010A Storage Pressure Meter. This system measures BP by recording the cuff pressure at which the interrupted blood flow returns to the tail. Before drug administration, training consisted of five sessions over 3 days. The SHRs were placed into a thermostatically controlled Lucite box of the Visitech system BP2010A, and heated on a heated pad maintained at 37 °C for 2 min before BP measurement started. The tail root of SHR was surrounded by an inflatable tubular sleeve, and a pulse electron detector was placed in the root of the tails. Then BP was measured after the computer provided a stable signal. The BP for the session was accepted as the average of five BP readings that were within 10 mm Hg. The average BP values were calculated by employing the ANOVA method.

In acute antihypertensive tests in vivo, the more potent and active compounds **7a** and **7g** were investigated further using spontaneously hypertensive rats (SHRs). Lisinopril was used as a positive control. The reduction in blood pressure (BP, systolic arterial pressure) of SHRs treated with compounds **7a** and **7g** at the same molar dose was slightly better than that of lisinopril under similar conditions. Additionally, the antihypertensive activity of **7a** and **7g** occurred evidently 0.5 h after oral treatment and lasted for four hours (Figure 4A,B). We then assessed the effects of compounds **7a** and **7g** in therapeutic antihypertensive tests in SHRs. We found that BP in compound **7a**- and **7g**-treated (100 mg/kg, Po. 7days) rats was decreased remarkably on the second day after oral administration. Then, the BP maintained at a relatively stable level. After drug withdrawal, the BP on the eighth day remained at a lower level. However, the BP began to rebound starting from the ninth day, and it returned back to the level before administration on the fourth day after drug withdrawal. Thus, compounds **7a** and **7g** showed remarkable hypotensive activity in SHRs (Figure 4C), and the maximum antihypertensive effect of the two compounds at a dose of 100 mg/kg daily was also approximately equivalent to that of lisinopril (20 mg/kg/day). It is worth noting that successive dosing has no effect on the body weight of the test rats in therapeutic antihypertensive tests.

## 4. Conclusions

In conclusion, we developed an efficient and shortcut method for drug discovery based on both the means of combinatorial chemistry and pharmaceutical design philosophy of traditional Chinese medicine. We call this method combination of traditional Chinese medicine chemistry (CTCMC). Using this method, and inspired by the “Danshen-borneol” drug pair and some classical ACE inhibitors, we designed eight novel peptidomimetics (phenolic acid-Pro-borneol) compounds, which were successively synthesized and evaluated in vitro and in vivo as potential ACE inhibitors with less side effects. Of these, bornyl esters of **7a** and **7g** exhibited good in vivo ACE-inhibitory activity. These two compounds are most likely acting as prodrugs since both were found to have moderate activity in vitro as inhibitors of ACE. Based on these preliminary and promising results, more suitable modifications in the phenolic moieties and amino acid residue adjoining proline are under development. This class of novel molecules, combined with their detailed antihypertensive activities, will be useful for future drug development and shall be presented in the near future.

## Figures and Tables

**Table 1:** ACE-inhibitory activity of each newly synthesized compound (IC_50_ values in μM).

Compound	Compound Structure	IC_50_ (μM)
**Lisinopril**	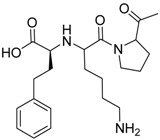	<0.01
**7a**	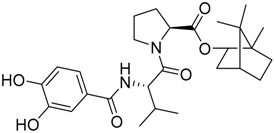	6.76 ± 0.96
**7b**	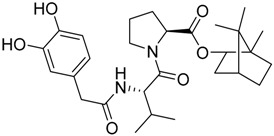	13.94 ± 1.61
**7c**	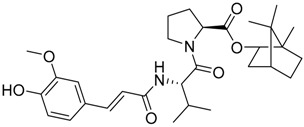	11.88 ± 1.82
**7d**	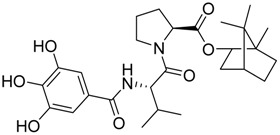	13.46 ± 2.02
**7e**	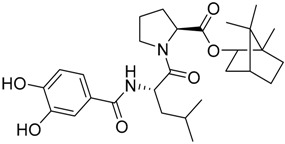	10.58 ± 1.72
**7f**	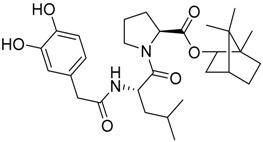	13.97 ± 1.84
**7g**	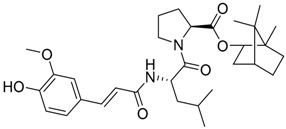	6.88 ± 0.85
**7h**	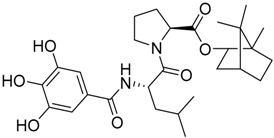	13.25 ± 2.47

Note: The amount of hippuric acid (HA) released is directly proportional to the ACE activity. Briefly, ACE activity was assayed by testing the release of HA from the hydrolysis of 0.3% (*w*/*v*) Hippuryl-l-Histidyl-l-Leucine Solution (HHL) (Sigma-aldrich, St. Louis, MO, USA, No. H1635). The enzyme reaction (0.33 unit/mL, 50 µL) was started by adding 50 µL of ACE enzyme, 200 µL of 50 mM HEPES HCl buffer with 300 mM sodium chloride (pH 8.3) and 50 µL HHL immediately mixed by swirling and incubated for 15 min at 37 °C. Then, 0.25 mL of 1 M HCl was added followed by 2 mL ethyl acetate (Sigma, No. 27, 052-0). The solution was shaken vigorously for 60 s and then centrifuged for 2 min. The clear upper layer (1.0 mL) was pipetted from each vial into corresponding 4-dram vials. The ethyl acetate was evaporated in a boiling water bath and 3 mL deionized water was added. The solution was mixed by inversion but not shaken. A spectrophotometer (U-3310) was used to record the absorbance of 228 nm.
