# Correction: Sun, Y.; et al. Design, Synthesis, and Evaluation of Novel 2-Hydroxypyrrolobenzodiazepine-5,11-dione Analogues as Potent Angiotensin Converting Enzyme (ACE) Inhibitors. *Molecules* 2017, 22, 1739

**DOI:** 10.3390/molecules23061438

**Published:** 2018-06-13

**Authors:** Ying Sun, Yujun Bai, Xirui He, Yajun Bai, Pei Liu, Zefeng Zhao, Xufei Chen, Xiaohui Zheng

**Affiliations:** 1 Key Laboratory of Resource Biology and Biotechnology in Western China, Ministry of Education, Northwest University, Xi’an 710069, China; syqsn1108@163.com (Y.S.); tianleyu@163.com (Y.B.); 6105194@163.com (X.H.); baiyj@nwu.edu.cn (Y.B.); lp183929@163.com (P.L.); zzf598155752@sina.com (Z.Z.); m13085333798@163.com (X.C.); 2 Key Laboratory of Synthetic and Natural Functional Molecule Chemistry of the Ministry of Education, College of Chemistry 0026 Materials Science, Northwest University, Xi’an 710127, China

The author wishes to make the following corrections to this paper [1]. Due to our oversight, the title of article [1] was same as that of article [2]. Because our research ideas and methods were different from those of article [2], we did not list it as a reference study in the article [1]. We would like to offer our sincere apologies to all of the authors of the article [2], who were Addla Dinesh, Jallapally Anvesh, Kanwal Abhinav, Sridhar Balasubramanian, Banerjee Sanjay K., and Kantevari Srinivas.

The corrections include: (1) replacing the title “Design, Synthesis, and Evaluation of Novel 2-Hydroxypyrrolobenzodiazepine-5,11-dione Analogues as Potent Angiotensin Converting Enzyme (ACE) Inhibitors” with “Design, Synthesis, and Evaluation of Novel Phenolic acid/Dipeptide/Borneol Hybrids as Potent Angiotensin Converting Enzyme (ACE) Inhibitors with Anti-hypertension Activity”; and (2) replacing “2-hydroxypyrrolobenzodiazepine-5,11-dione analogues” with “phenolic acid/dipeptide/borneol hybrids” in the abstract.

The authors would like to apologize for any inconvenience caused to the readers by these changes.
